# How many markers are needed to robustly determine a cell’s type?

**DOI:** 10.1016/j.isci.2021.103378

**Published:** 2022-01-10

**Authors:** Stephan Fischer, Jesse Gillis

## Main text

(iScience *24*, 103292-1–103292-23; November 19, 2021)

During the preparation of the revised version of the manuscript, the supplemental files containing the markers genes for BICCN cell types were duplicated. The authors have now provided the correct version of Data S2 and S3 containing markers for cell types of increasing granularity (class, subclass, cluster). The authors apologize for any confusion to the readers.

## Supplemental information

Data S2. Subclass-level markers, related to STAR Methods

Data S3. Cluster-level markers, related to STAR Methods

